# No Evidence for Infection of UK Prostate Cancer Patients with XMRV, BK Virus, *Trichomonas vaginalis* or Human Papilloma Viruses

**DOI:** 10.1371/journal.pone.0034221

**Published:** 2012-03-28

**Authors:** Harriet C. T. Groom, Anne Y. Warren, David E. Neal, Kate N. Bishop

**Affiliations:** 1 Division of Virology, MRC National Institute for Medical Research, London, United Kingdom; 2 Department of Pathology, University of Cambridge, Cambridge, United Kingdom; 3 Department of Oncology, University of Cambridge, Cambridge, United Kingdom; McGill University AIDS Centre, Canada

## Abstract

The prevalence of specific infections in UK prostate cancer patients was investigated. Serum from 84 patients and 62 controls was tested for neutralisation of xenotropic murine leukaemia virus-related virus (XMRV) Envelope. No reactivity was found in the patient samples. In addition, a further 100 prostate DNA samples were tested for XMRV, BK virus, *Trichomonas vaginalis* and human papilloma viruses by nucleic acid detection techniques. Despite demonstrating DNA integrity and assay sensitivity, we failed to detect the presence of any of these agents in DNA samples, bar one sample that was weakly positive for HPV16. Therefore we conclude that these infections are absent in this typical cohort of men with prostate cancer.

## Introduction

Prostate cancer (PC) contributes significantly to the global disease burden due to cancer and is the most common cancer in men in the UK (http://info.cancerresearchuk.org/cancerstats). Current screening methods and patient management decisions are hindered by a fundamental lack of insight into the natural history of the disease, which is likely to be multi-factorial. Researchers proposed an infectious cause as early as the 1950s and since then various pathogens have been associated with the disease (Reviewed in [1]). Although epidemiological studies indicate that a history of acquiring sexually transmitted diseases increases the risk of PC, no definitive association with a specific infection has been shown. We decided to concurrently examine the prevalence of four different infectious agents that have each independently been associated with PC. We aimed to increase the chances of detection by studying samples from a well-characterised cohort of patients with severe PC.

Firstly, we looked for the new gammaretrovirus, xenotropic murine leukaemia virus (MLV)-related virus (XMRV), that was isolated from familial PC patient tissue in 2006 [2]. A second, larger, controlled study in 2009 suggested that XMRV was associated with high-grade tumours and was not limited to familial PC cases [3]. However, several subsequent studies have found no link with XMRV and this association has remained controversial. Whilst this manuscript was in preparation a study by Paprotka *et al.* demonstrated the likely recombinant origin of XMRV [4]; these ancestral viruses have since been further characterised [5]. Recently, Martinez-Fierro and colleagues detected human papilloma viruses (HPV) in 20% of PC cases by PCR [6]. High risk HPVs are causative agents in cervical cancer and their E6 and 7 proteins are able to immortalise prostate cells [7]. Polyomaviruses also have the potential for carcinogenesis and BK virus (BKV) has been frequently detected in PC specimens, most recently in tissue, urine and plasma [8], [9]. Finally, the parasite *Trichomonas vaginalis* (TV) is known to cause prostatitis and showed a slightly increased prevalence rate in PC in a recent case-control study [10]. Therefore these three pathogens were investigated in addition to XMRV.

We examined PC tissue DNA for XMRV, BKV, TV and HPV nucleic acids as well as testing PC patient sera for the presence of neutralising antibodies to XMRV Env. Despite proving the assays were highly sensitive, none of the patient samples were positive for infection, with the exception of one HPV-positive DNA sample. Our results therefore suggest that there is no association with any of the agents and PC and that clinical approaches should not focus on counteracting these infectious agents in men.

## Results

**Figure 1:**
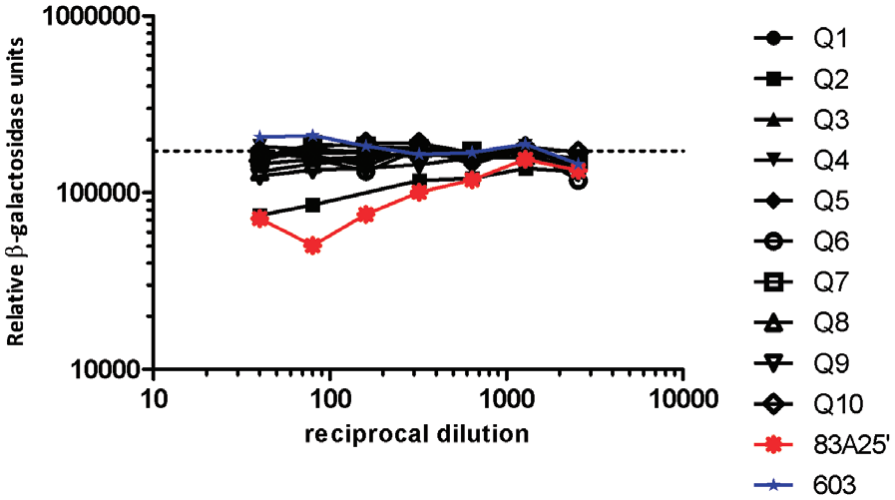
Detection of XMRV neutralising antibodies in human sera. Infectivity of XMRV after incubation with patient serum or monoclonal suspension. Infectivity (measured as relative β-galactosidase units) is plotted against the reciprocal of the serum dilution (black symbols) or monoclonal suspension (blue data points, monoclonal 603, negative control; red data points, 83A25′, positive control). Dashed line indicates level of infectivity in the absence of serum/monoclonal. Q1–10 are patient designations. Q2 exhibits neutralisation.

Eighty-four UK PC patient and 62 control sera (from men with low prostate-specific antigen (PSA) or high PSA with a negative biopsy) were tested for the presence of anti-XMRV antibodies using a neutralisation assay that has been previously described [11]. Despite validated monoclonal antibody controls showing reproducible neutralising activity in this assay, none of the PC patient sera showed any evidence of anti-XMRV antibodies. One serum sample from a control, Q2, did show specific reactivity against XMRV at high serum concentrations (Figure 1).

**Figure 2:**
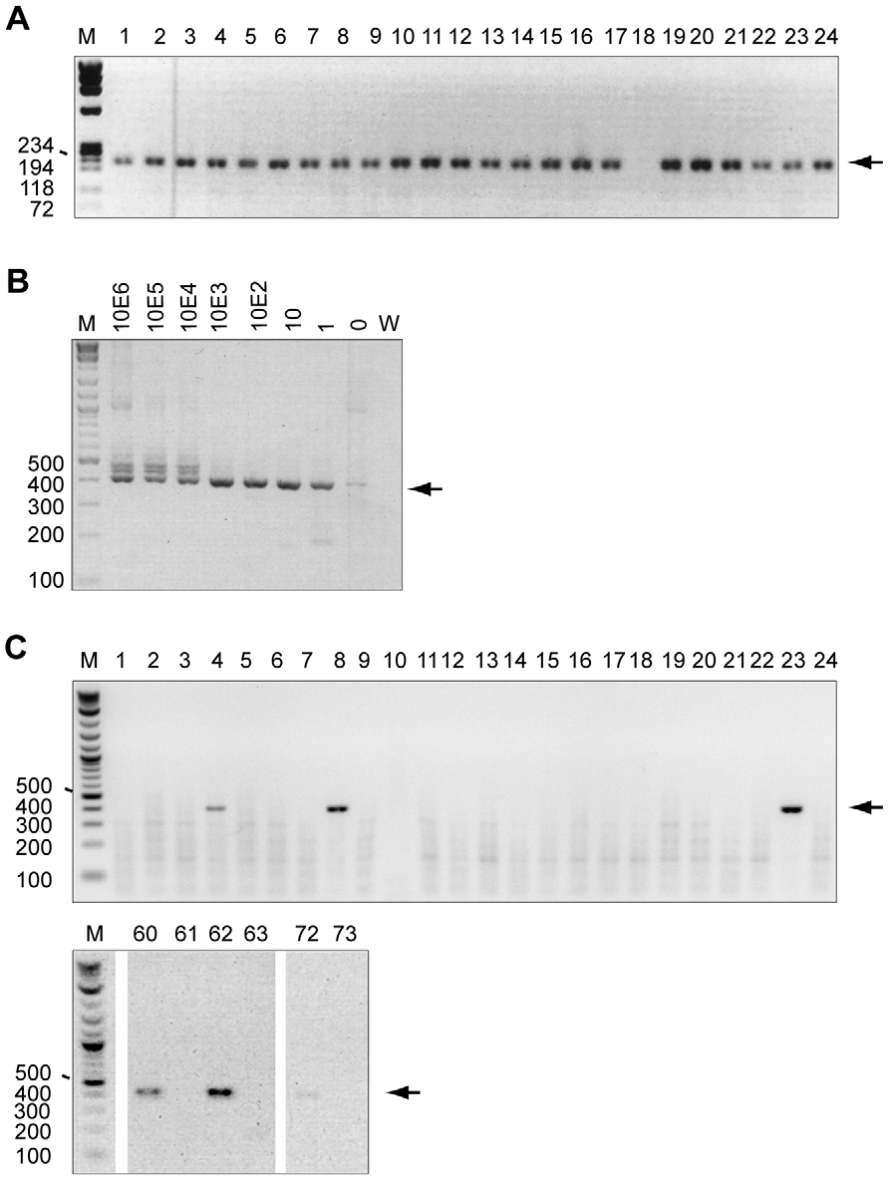
DNA integrity and detection of XMRV sequences in prostate cancer tissue DNA. Panels A-C show 1% agarose gels stained with ethidium bromide. For all, M indicates marker, W indicates water, numbers on the left indicate size of markers in base pairs, arrows indicate expected band size for each PCR. A) Detection of *hgapdh* in DNA isolated from PC tissue DNA samples 1–24 using single round PCR. Expected PCR product size, 225 base pairs. B) Standard curve to determine the detection limit of XMRV *gag* nested PCR. Ten-fold serial dilutions from 10^6^ to 1 molecule per µl of XMRV plasmid (pcDNA3.1/VP62) were made in a background of an excess of human genomic DNA. Second round amplification products of the *gag* nested PCR are shown. Numbers above lanes indicate input number of molecules. Expected PCR product size 413 base pairs. Sequencing the slower migrating faint band in the “0” lane (human genomic DNA alone) showed this to be non-specific amplification of human genes (data not shown). C) Detection of XMRV *gag* sequences in DNA isolated from prostate cancer tissue samples. Second round amplification products from a subset of patients are shown as in (B). Samples 4, 8 23, 60, 62 and 72 are positive.

**Figure 3:**
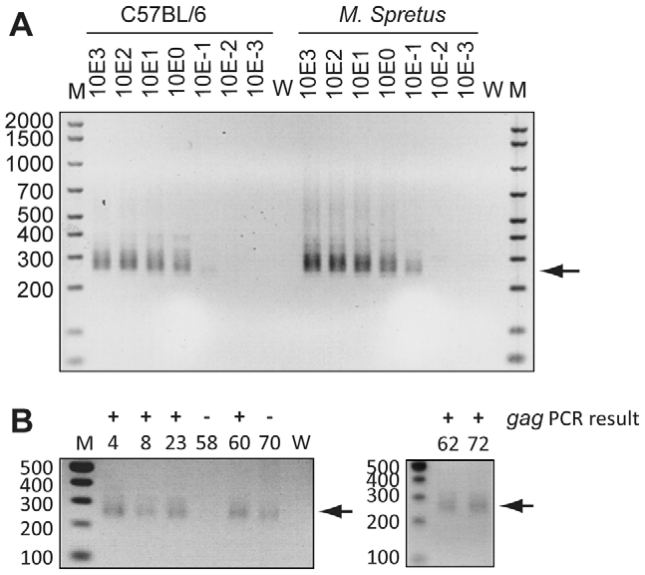
Detection of murine DNA contamination in prostate cancer tissue DNA. Panels A and B show 1% agarose gels stained with ethidium bromide. For all, M indicates marker, numbers on the left indicate size of markers in base pairs, W indicates water, arrows indicate expected band size for each PCR. Products appear as a smear due to varying amplicon lengths. For reference, there are approximately one thousand IAP copies per mouse genome. A) Intra-cisternal A-type particle DNA was amplified from serial dilutions of mouse DNA (0.001 to 10^3^ genome equivalents, either C56BL/6 or *Mus Spretus*). Products from single round amplification are shown. Numbers above lanes indicate number of mouse genome equivalents in the PCR reaction. B) Products from PCR amplification of intra-cisternal A-type particle DNA from selected PC tissue DNA samples. Numbers above lanes indicate patient designations. Samples 4, 8, 23, 60, 62, 70 and 72 are positive. +/− indicate positive or negative result in *gag* PCR (Figure 2).

Unfortunately, DNA was not available from the same cohort of patients tested above and so DNA was extracted from a further one hundred prostate cancer tissue samples from another similarly well-characterised cohort. DNA samples were tested for integrity by *hgapdh* PCR and 96 showed detectable product after a single round (a subset is shown in Figure 2A). The four samples that failed to amplify product were included in further tests as controls for assay contamination. Extracted DNA was tested for XMRV using a nested PCR amplifying the viral *gag* gene as previously described [2]. This PCR reliably detects single copies of plasmid control diluted in an excess of human DNA in every experiment (Figure 2B). This PCR is also capable of detecting related gammaretroviruses (MLVs), including those found as endogenous viruses in mice. Six out of 100 PC DNA samples gave a product of the expected size (Figure 2C). As XMRV is related to endogenous retroviruses present in mice, the presence of contaminating murine DNA could lead to a positive result in this PCR. To control for this, positive samples were also tested for murine DNA using a PCR specific for the murine endogenous retroviral element intracisternal A particles (IAP) [12]. This PCR can detect low levels of mouse DNA due to the high frequency of IAPs in mouse genomes (Figure 3A). All six samples that were positive for XMRV *gag* were also positive using the IAP PCR (Figure 3B, Tables 1 and S1). To clarify the origin of these bands more precisely, the amplicons were cloned and sequenced. Phylogenetic analysis of sequenced “*gag*” amplicons supported the assertion that they were derived from contaminating murine nucleic acid (Figure S1).

**Table 1:** Summary of nucleic acid detection results.

	Nucleic acid detected (amplicon size, bp)						
Sample	*Hgapdh* control (225)	XMRV *gag* (413)	IAP (approx. 280)	BKV (453)	TV (108)	hDNA control (na)	HPV (65)
4	+	+	+	−	−	+	−
8	+	+	+	−	−	+	−
18	−	−	nd	−	−	−	−
23	+	+	+	−	−	+	−
25	−	−	nd	−	−	−	−
26	−	−	nd	−	−	−	−
58	+	−	−	−	−	+	−
59	+	−	nd	−	−	−	−
60	+	+	+	−	−	+	−
62	+	+	+	−	−	+	−
63	+	−	nd	−	−	−	−
65	−	−	nd	−	−	+	−
68	+	−	nd	−	−	−	−
69	+	−	nd	−	−	−	−
70	+	−	+	−	−	+	−
72	+	+	+	−	−	+	−
73	+	−	nd	−	−	−	−
74	+	−	nd	−	−	−	−
75	+	−	nd	−	−	+	+
84	+	−	nd	−	−	−	−
87	+	−	nd	−	−	−	−
92	+	−	nd	−	−	−	−
95	+	−	nd	−	−	−	−
**Modal results**	**+**	**−**	**nd**	**−**	**−**	**+**	**−**
**TOTAL**	**96/100**	**6/100**	**6/6 ( gag +)**	**0/100**	**0/100**	**87/100**	**1/100**
			**1/2 (gag −)**				

Individual samples with results differing from the mode result set are shown. Modal results are shown in the penultimate row. Totals are given at the end of each column. +, positive result, −, negative result, nd, not determined. *Hgapdh*, human glyceraldehyde 3-phosphate dehydrogenase, XMRV, xenotropic murine leukaemia virus-related virus, IAP, intra-cisternal A particle, BKV, BK virus, TV, *Trichomonas vaginalis*, hDNA, human DNA, HPV, human papilloma virus.

**Figure 4:**
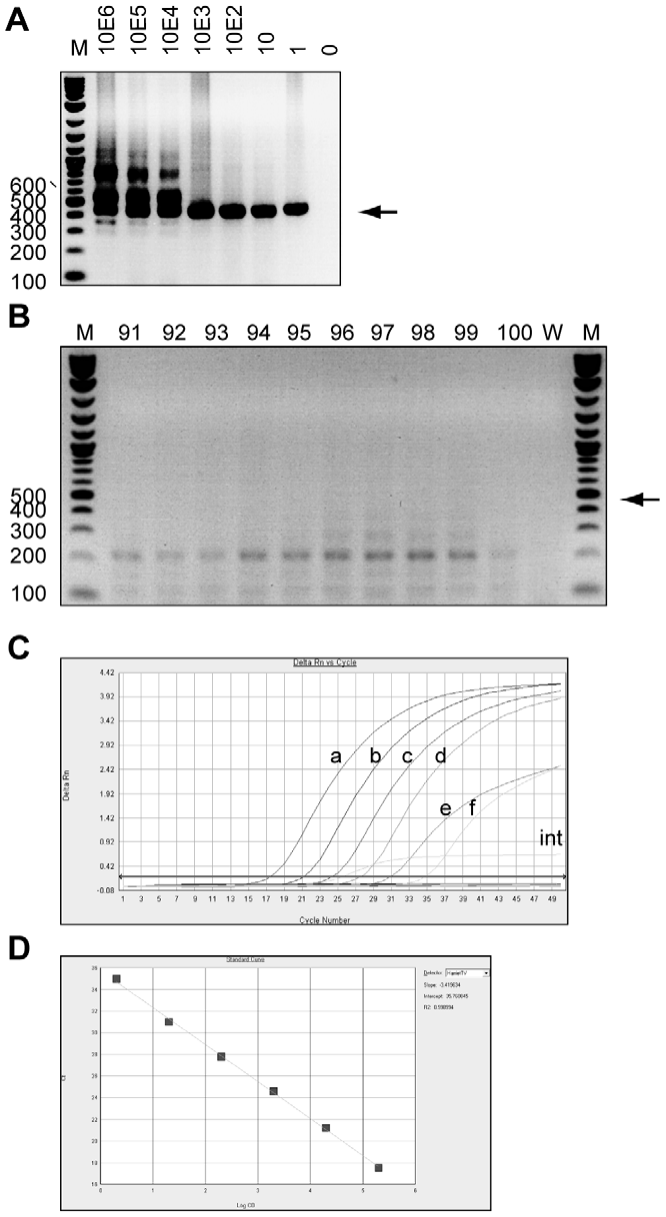
Detection of BKV and TV in prostate cancer tissue DNA. Panels A and B show 1% agarose gels stained with ethidium bromide. For both, M indicates marker, numbers on the left indicate size of markers in base pairs, arrows indicate bands of the expected size for the second round (453 bp). A) Serial dilutions of BKV plasmid (pBR322-Dunlop) were made from 10^6^ to 1 molecule(s) and amplified by nested PCR. Products of the second round of amplification are shown. Numbers above lanes indicate number of molecules in the PCR reaction. B) Products from the second round of nested PCR amplification of BKV from a subset of PC tissue DNA samples. Numbers above lanes indicate patient designations. C) Amplification plot showing curves for quantitative PCR reactions detecting TV using Quantification of Trichomonas vaginalis Advanced Kit. Serial dilutions of positive control are shown as well as lack of amplification for PC tissue DNA samples 1–50 (no amplification and thus all below the threshold line). Inputs were as follows: int, internal extraction control (detected on a separate filter), a, 10^6^ TV molecules, b, 10^5^ TV molecules, c, 10^4^ TV molecules, d, 10^3^ TV molecules, e, 10^2^ TV molecules, f, 10 TV molecules. Plot shows delta Rn against cycle number. D) Plot of Ct values against log of positive control input concentration demonstrating linearity, R^2^>0.99.

**Figure 5:**
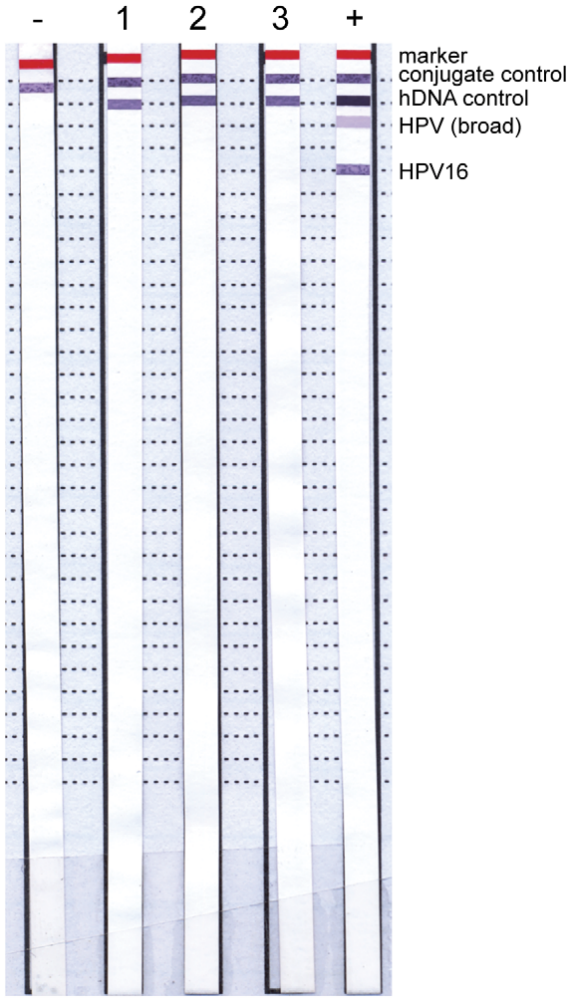
Detection of HPV in prostate cancer tissue DNA. HPV DNA in PC tissue DNA samples was detected using INNO-LiPA HPV Genotyping Extra. HPV DNA was amplified from samples using primers to conserved sequences generating biotinylated products. Biotinylated amplicons were then hybridised to strips bearing conserved and type-specific HPV probes. Bands indicate binding of the biotinylated sample DNA to the target indicated. Marker is for aligning strips, conjugate control confirms the kit reagents are functioning correctly, hDNA control indicates the presence of human DNA in the sample, HPV (broad) indicates binding of HPV DNA to probes that bind conserved regions. All other dotted lines show areas bound by specific HPVs e.g. HPV16 (shown). Numbers above strips are patient designations (only 3 sample strips are shown for clarity), −, negative control (water), +, positive control (supplied with kit). Result for the single HPV positive is not shown as it was too faint to appear on a scanned image.

DNA samples were also tested for the presence of BKV by nested PCR. Despite reliable detection of one molecule of BKV plasmid in repeated attempts (pBR322-Dunlop, Figure 4A), none of the patient samples gave a band of the appropriate size (subset shown in Figure 4B). Similarly, patient samples were negative for TV using a commercially available real-time PCR testing kit (Figure 4C). This assay was capable of reliably detecting ten copies of TV (Figure 4C+D). These negative results were not due to a failure of the extraction procedure as an internal extraction control was successfully amplified. All DNA samples were then tested for HPV and all samples but one, which had weak hybridisation to HPV16, were found to be negative, despite the successful amplification of human DNA using the kit control probe in all but one sample (Samples 1–3 shown in Figure 5). A summary of these results for all 100 PC DNA samples is given in Tables 1 and S1.

## Discussion

An extensive and controversial literature exists linking infectious agents to PC, particularly XMRV and HPV [1], [13]. As we had access to a large, well-characterised cohort of PC patients and experience using molecular techniques to detect viruses, we felt it would be useful to investigate the presence of four agents; XMRV, BKV, TV and HPV, in the same PC samples.

In recent years there has been much interest and controversy in the association of XMRV with PC. In an assay developed to detect an immune response to XMRV, one serum sample in our study was able to neutralise the virus (Q2, Figure 1). However, this serum was from a low PSA control individual and therefore there was no association with PC. This neutralisation assay was similar to that used by Arnold *et al.*, except that the pseudotyped virus-like particles used were based on MLV rather than HIV [14]. This conceivably represents a false positive result due to epitope cross-reactivity. Regrettably, neither further serum for testing reactivity by western blot nor DNA for testing by PCR were available for this individual. Furthermore, we did not find any evidence for the canonical XMRV [2] in PC DNA samples by PCR. Six samples amplified a product in the *gag* PCR but were also positive for murine DNA, suggesting that this PCR had amplified endogenous murine retroviral sequences that are present in high copy numbers in mice (Figures 2 and 3). Phylogenetic analysis of amplicons supports this hypothesis (Figure S1). Additionally, one *gag* negative sample was positive in the IAP PCR, showing that the IAP PCR is capable of amplifying contaminating murine DNA that was not detected by the *gag* PCR.

Together, our serological and PCR data show no association of gammaretroviruses and PC. This agrees with recent genetic evidence determining the origin of XMRV that indicates that XMRV-related viruses are not actually human pathogens [4], reviewed in [15]. Despite making every effort to isolate DNA and perform assays in mouse-free facilities using new equipment bought for this study, our IAP PCR results highlight the problem of contamination of samples with murine DNA. Indeed, it has recently been reported that previously positive samples may be due to contamination [16], [17], [18]. Contamination is an issue widely discussed in the XMRV literature [13]. Researchers using extremely sensitive nucleic acid techniques to detect pathogens in patient samples should be wary of misinterpreting apparent positive results [19]. In the case of XMRV, several methods of contamination are possible. Sequencing of PCR products is essential, together with checking for the presence of murine DNA that could give rise to products in PCR due to the high level of endogenous retroviruses. However, highly related endogenous sequences or contaminating plasmid DNA might not be flagged by this method. Specific PCR assays designed to detect these contaminants must be used [20]. Viral contaminants or contamination with laboratory-derived infected human cells are impossible to differentiate from *bona fide* human infection. The only way to confirm an apparently positive result is by independently reproducing it, which is why it is important for several groups to conduct their own studies. Whilst this manuscript was in preparation two key papers citing detection of XMRV in chronic fatigue syndrome patient samples were retracted in the light of evidence for contamination [21], [22], [23], [24].

We also utilised PCR assays to screen our patient samples for three pathogens previously linked to PC. We used published primers reported to detect BKV in PC samples [25], and two commercial kits to look for TV and HPV. The TV kit is designed to have the broadest detection profile possible whilst remaining specific to the TV genome. The HPV assay is already used to monitor cervical tissue samples and can identify 28 types of HPV, including all currently known high-risk and probable high-risk genotypes as well as a number of low-risk types. If an association was found with PC and any of these pathogens then these assays would provide a rapid, simple way to screen patients and identify those for whom anti-pathogen treatments may be beneficial. All the assays were validated using positive controls (Figure 4A, C and D) and the quality of DNA extracted from PC samples was assessed by amplification of human genes (Figures 2 and 5, Tables 1 and S1). However, none of the samples tested positive for BKV or TV and only one sample tested positive for HPV (type 16). This suggests that these pathogens are not common in PC tissue and thus treatment for infections is unlikely to assist patients in general.

Although a negative result is difficult to prove conclusively, we would argue that our DNA samples were of sufficient quality to amplify human control genes and that amplification of the internal control indicated that the extraction procedure did not inhibit PCR. It is possible that the process of formalin-fixing and paraffin embedding led to fragmentation of the DNA. If this were the case then the *hgapdh* control could only confirm the possible detection of amplicons shorter than 225 bp (HPV and TV). However, if the prevalence of XMRV or BKV were high, one would still expect to detect the viral sequences in some of the samples, despite random fragmentation. Additionally, the XMRV, BKV and TV assays were capable of detecting one-to-ten genome equivalents implying the assays used were exceedingly sensitive. These detection limits were reliably observed on repeated attempts. Our data support the conclusions of Sfanos *et al.* who examined the prevalence of these agents in PC material as part of a larger study focussed on the detection of bacterial infection of the prostate [26]. These authors were also unable to detect any of these pathogens in PC patients with the exception of one positive BKV result.

Studies that found pathogen DNA in prostate tissue may reflect contamination of the prostate with pathogens associated with the proximal tissues or even non-human sources. The historical association of PC with sexually transmitted infection could reflect increased inflammation due to infection but may not require infection with a particular pathogen. Alternatively, this study may not have included the most relevant pathogen or be large enough to reveal the small contribution of each of the agents investigated. A generally reduced exposure to sexually-transmitted infections in recent times, in combination with the relatively small numbers in our study, may preclude detection of infected individuals. However, the cohort examined is a group of well characterised patients with severe disease and if any of the above infections were strongly associated with PC, and therefore clinically relevant, one would expect to have detected them in this study. We therefore conclude that XMRV, BKV, TV and HPV are not prevalent in this PC cohort and suggest that alternative aetiologies be considered for PC.

## Materials and Methods

### Ethics statement

Patient samples were collected through the ProMPT study with ethical approval from the Trent Multi-centre Research Ethics Committee and with informed, written consent.

### Plasmids

HG1 is a replication incompetent derivative of pcDNA3.1/VP62 with deletions in the promoter region and packaging signal [27]. pLTR-LacZ is an MLV-based retroviral vector encoding β-galactosidase [27]. The pBR322-Dunlop plasmid (ATCC #45025) was received from Mike Imperiale (Ann Arbor) and has been previously described [28].

### Sample collection and processing

Serum samples from four groups of patients were analysed: 30 low PSA controls, 32 high PSA but negative biopsy controls and 84 PC patients. DNA was isolated from 100 formalin-fixed paraffin-embedded PC slices using the QIAamp DNA FFPE Tissue Kit (Qiagen) according to the manufacturer's instructions, except samples were incubated in lysis buffer overnight to ensure complete lysis. DNA was eluted in 50 µl elution buffer and the concentration determined using a NanoDrop spectrophotometer.

### Detection of neutralising XMRV antibodies in serum

Neutralisation assays using XMRV have been previously described [11]. Monoclonal antibodies 83A25′ and 603 were used as positive and negative controls respectively, and cells were monitored for viability by visual inspection at the time of harvesting.

### PCR

Sequences of all primers have been previously described as cited and are given in Table 2. Single round PCR detection of *hgapdh* and nested PCR for XMRV *gag* were carried out as described in [2] using primers hGAPDH-66F and hGAPDH-291R and GAG-O-F, GAG-O-R, GAG-I-F and GAG-I-R. IAP DNA single round PCRs were carried out described in [12] and detection of BKV was carried out by nested PCR as previously described [25] using primers BKV_for_, BKV_rev_, BKVnes_for_ and BKVnes_rev_. To determine sensitivity, 10-fold serial dilutions from 1 to 10^6^ molecules of pcDNA3.1/VP62 for XMRV, C56BL/6 or *Mus Spretus* DNA for IAP or pBR322-Dunlop for BKV, were tested using the above PCRs. The products of all single round/nested PCRs were analysed by gel electrophoresis. For the detection of *Trichomonas vaginalis*, samples were tested using the Quantification of Trichomonas vaginalis Advanced Kit (PrimerDesign Ltd) according to the manufacturer's instructions using the Mini Precision Low-Rox mastermix (PrimerDesign) and the ABI 7500 Real-Time PCR System (Applied Biosystems). Internal DNA extraction controls were used for all detection methods alongside a positive control standard curve and negative controls appropriate for each assay. Amplification of HPV DNA for typing was carried out using the INNO-LiPA HPV genotyping Extra Amp kit according to the manufacturer's instructions. Typing was then carried out using the INNO-LiPA HPV genotyping Extra kit on an *Auto*-LiPA instrument according to the manufacturer's instructions (all INNOgenetics).

**Table 2:** Primers used in this study.

Target	Forward primer (5′-3′)	Reverse primer (5′-3′)
*hgapdh*	GAAGGTGAAGGTCGGAGTC	GAAGATGGTGATGGGATTTC
XMRV gag (outer)	CGCGTCTGATTTGTTTTGTT	CCGCCTCTTCTTCATTGTTC
XMRV gag (inner)	TCTCGAGATCATGGGACAGA	AGAGGGTAAGGGCAGGGTAA
IAP	ATAATCTGCGCATGAGCCAAGG	AGGAAGAACACCACAGACCAGA
BKV (outer)	TTTTGGAACCTGGAGTAGCTCAGAGGTTT	GCTTGACTAAGAAACTGGTGTAGAT
BKV (inner)	CCTCTTTGCCCAGATACCCTGTACT	GAGAATCTGCTGTTGCTTCTTCATC
Amplicon sequencing	GATCCTTTGATTTTCTACCGAAG	CTTACGTGCCGATCAAGTCA

*Hgapdh*, human glyceraldehyde 3-phosphate dehydrogenase, XMRV, xenotropic murine leukaemia virus-related virus, IAP, intra-cisternal A particle, BKV, BK virus. Outer and inner primer sets given for nested PCRs.

### Amplicon analysis

PCR products were cloned into the pSMART® HCKan vector using the CloneSmart HCKan Blunt Cloning Kit (Lucigen) according to the manufacturer's instructions. Ligated pSMART vectors were sequenced using primers shown in Table 2. Sequences were aligned to the appropriate region of *gag* in XMRV sequences available on PubMed and relevant MLV sequences using MegAlign (DNASTAR LaserGene 8). Sequences have been deposited in GenBank (JQ048948–JQ048955). Further details of sequences and accession numbers are given in the figure legend. Model selection for phylogenetic analysis was carried out using Modelgenerator v8.5 [29] and Bayesian phylogenies were predicted using the general time reversible model of nucleotide substitution and a gamma-distributed rate heterogeneity using the program MrBayes [30]. The MCMC algorithm was run for 4,000,000 generations, sampling trees every 100 generations. ESS values were calculated using Tracer (http://beast.bio.ed.ac.uk/Tracer). The consensus tree with branch lengths and posterior probability support values for the internal nodes was visualised in FigTree (http://tree.bio.ed.ac.uk/software/figtree), rooting on MoMLV as an outgroup and edited using Adobe Illustrator.

## Supporting Information

Figure S1 **Phylogenetic analysis of** ***gag*** **sequence amplicons.** Amplicons from *gag* nested PCR (Figure 2) were cloned, sequenced and compared to the same region from known XMRV and MLV sequences. Identical sequences in the initial panel were removed from the phylogenetic analysis to prevent bias. Accession numbers are as follows: PreXMRV1 (Likely XMRV ancestral sequence, NC_007815.2), murine C-type retrovirus (X94150), AKV (endogenous ecotropic MLV, J01998), DG-75 (xenoptropic Moloney MLV variant, AF221065), murine AIDS virus (S80082), 22Rv1 (XMRV derived from 22Rv1 cells, FN692043.2), VP62 (original XMRV isolate from a PC patient, DQ399707). Xmv, Mpmv and Pmv (xenoptropic, modified polytropic and polytropic endogenous MLV) sequences were obtained from Jern *et al. PLoS Genet*, 2007. FB579966 and FJ907198 are reported isolates from a patient and 22Rv1 cells respectively. Bayesian phylogenies were predicted using the general time reversible model of nucleotide substitution and a gamma-distributed rate heterogeneity using the program MrBayes. The MCMC algorithm was run for 4,000,000 generations for two simultaneous independent analyses, sampling trees every 100 generations. At the end of the analysis PSRF = 1.000 and standard deviation = 0. ESS values were 899 and 959 for the two analyses (determined using Tracer v1.5). The consensus tree is shown with branch lengths and posterior probability support values for the internal nodes visualised in FigTree v1.3.1, rooting on Moloney MLV as an outgroup. Sequenced amplicons are shown in blue. These sequences have been deposited in GenBank (JQ048948–JQ048955). The originally described XMRV sequence, VP62, is shown in red. Support values, and therefore confidence, is limited by lack of divergence in *gag*, however clustering of amplicons with endogenous retroviral sequences is supportive of their being derived from contaminating murine DNA.
(TIF)

Table S1 Full nucleic acid detection results.
(DOC)
