# Supplementary figures and images for: No Evidence for Infection of UK Prostate Cancer Patients with XMRV, BK Virus, Trichomonas vaginalis or Human Papilloma Viruses
Source: PLoS One. 2012 Mar 28;7(3):e34221. doi: 10.1371/journal.pone.0034221 (PMC3314598; doi:10.1371/journal.pone.0034221)

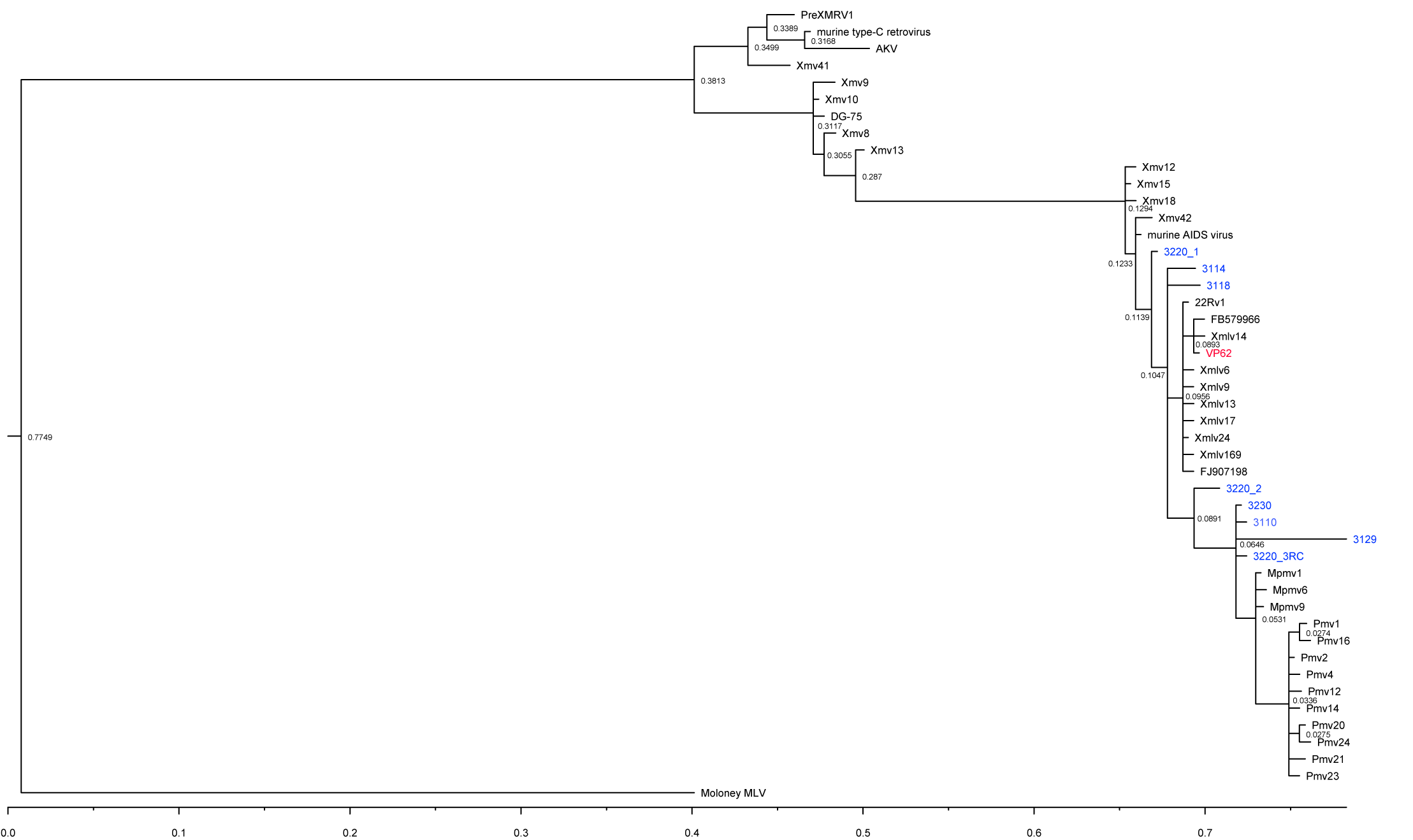

Supplement: Figure S1 — Phylogenetic analysis of gag sequence amplicons. Amplicons from gag nested PCR (Figure 2) were cloned, sequenced and compared to the same region from known XMRV and MLV sequences. Identical sequences in the initial panel were removed from the phylogenetic analysis to prevent bias. Accession numbers are as follows: PreXMRV1 (Likely XMRV ancestral sequence, NC_007815.2), murine C-type retrovirus (X94150), AKV (endogenous ecotropic MLV, J01998), DG-75 (xenoptropic Moloney MLV variant, AF221065), murine AIDS virus (S80082), 22Rv1 (XMRV derived from 22Rv1 cells, FN692043.2), VP62 (original XMRV isolate from a PC patient, DQ399707). Xmv, Mpmv and Pmv (xenoptropic, modified polytropic and polytropic endogenous MLV) sequences were obtained from Jern et al. PLoS Genet, 2007. FB579966 and FJ907198 are reported isolates from a patient and 22Rv1 cells respectively. Bayesian phylogenies were predicted using the general time reversible model of nucleotide substitution and a gamma-distributed rate heterogeneity using the program MrBayes. The MCMC algorithm was run for 4,000,000 generations for two simultaneous independent analyses, sampling trees every 100 generations. At the end of the analysis PSRF = 1.000 and standard deviation = 0. ESS values were 899 and 959 for the two analyses (determined using Tracer v1.5). The consensus tree is shown with branch lengths and posterior probability support values for the internal nodes visualised in FigTree v1.3.1, rooting on Moloney MLV as an outgroup. Sequenced amplicons are shown in blue. These sequences have been deposited in GenBank (JQ048948–JQ048955). The originally described XMRV sequence, VP62, is shown in red. Support values, and therefore confidence, is limited by lack of divergence in gag, however clustering of amplicons with endogenous retroviral sequences is supportive of their being derived from contaminating murine DNA. (TIF) [file pone.0034221.s001.tif]
