# Supplementary material for: No Evidence for Infection of UK Prostate Cancer Patients with XMRV, BK Virus, Trichomonas vaginalis or Human Papilloma Viruses
Source: PLoS One. 2012 Mar 28;7(3):e34221. doi: 10.1371/journal.pone.0034221 (PMC3314598; doi:10.1371/journal.pone.0034221)
Supplement: Table S1 — Full nucleic acid detection results. (DOC) [file pone.0034221.s002.doc]

**Table S1: Full** nucleic acid detection results

|  | **Nucleic acid detected (amplicon size, bp)** | | | | | | |
| --- | --- | --- | --- | --- | --- | --- | --- |
| **Sample** | ***Hgapdh control*** (225) | ***XMRV gag*** (413) | **IAP** (approx. 280) | **BKV** (453) | **TV** (108) | **hDNA control** (na) | **HPV** (65) |
| 1 | + | − | nd | − | − | + | − |
| 2 | + | − | nd | − | − | + | − |
| 3 | + | − | nd | − | − | + | − |
| **4** | **+** | **+** | **+** | **−** | **−** | **+** | **−** |
| 5 | + | − | nd | − | − | + | − |
| 6 | + | − | nd | − | − | + | − |
| 7 | + | − | nd | − | − | + | − |
| **8** | **+** | **+** | **+** | **−** | **−** | **+** | **−** |
| 9 | + | − | nd | − | − | + | − |
| 10 | + | − | nd | − | − | + | − |
| 11 | + | − | nd | − | − | + | − |
| 12 | + | − | nd | − | − | + | − |
| 13 | + | − | nd | − | − | + | − |
| 14 | + | − | nd | − | − | + | − |
| 15 | + | − | nd | − | − | + | − |
| 16 | + | − | nd | − | − | + | − |
| 17 | + | − | nd | − | − | + | − |
| **18** | **−** | **−** | **nd** | **−** | **−** | **−** | **−** |
| 19 | + | − | nd | − | − | + | − |
| 20 | + | − | nd | − | − | + | − |
| 21 | + | − | nd | − | − | + | − |
| 22 | + | − | nd | − | − | + | − |
| **23** | **+** | **+** | **+** | **−** | **−** | **+** | **−** |
| 24 | + | − | nd | − | − | + | **−** |
| **25** | **−** | **−** | **nd** | **−** | **−** | **−** | **−** |
| **26** | **−** | **−** | **nd** | **−** | **−** | **−** | **−** |
| 27 | + | − | nd | − | − | + | − |
| 28 | + | − | nd | − | − | + | **−** |
| 29 | + | − | nd | − | − | + | − |
| 30 | + | − | nd | − | − | + | − |
| 31 | + | − | nd | − | − | + | − |
| 32 | + | − | nd | − | − | + | − |
| 33 | + | − | nd | − | − | + | − |
| 34 | + | − | nd | − | − | + | − |
| 35 | + | − | nd | − | − | + | − |
| 36 | + | − | nd | − | − | + | − |
| 37 | + | − | nd | − | − | + | − |
| 38 | + | − | nd | − | − | + | **−** |
| 39 | + | − | nd | − | − | + | − |
| 40 | + | − | nd | − | − | + | − |
| 41 | + | − | nd | − | − | + | − |
| 42 | + | − | nd | − | − | + | − |
| 43 | + | − | nd | − | − | + | − |
| 44 | + | − | nd | − | − | + | − |
| 45 | + | − | nd | − | − | + | **−** |
| 46 | + | − | nd | − | − | + | − |
| 47 | + | − | nd | − | − | + | − |
| 48 | + | − | nd | − | − | + | − |
| 49 | + | − | nd | − | − | + | **−** |
| 50 | + | − | nd | − | − | + | − |
| 51 | + | − | nd | − | − | + | − |
| 52 | + | − | nd | − | − | + | − |
| 53 | + | − | nd | − | − | + | − |
| 54 | + | − | nd | − | − | + | − |
| 55 | + | − | nd | − | − | + | − |
| 56 | + | − | nd | − | − | + | − |
| 57 | + | − | nd | − | − | + | − |
| **58** | **+** | **−** | **−** | **−** | **−** | **+** | **−** |
| **59** | **+** | **−** | **nd** | **−** | **−** | **−** | **−** |
| **60** | **+** | **+** | **+** | **−** | **−** | **−** | **−** |
| 61 | + | − | nd | − | − | + | − |
| **62** | **+** | **+** | **+** | **−** | **−** | **+** | **−** |
| **63** | **+** | **−** | **nd** | **−** | **−** | **−** | **−** |
| 64 | + | − | nd | − | − | − | **−** |
| **65** | **−** | **−** | **nd** | **−** | **−** | **+** | **−** |
| 66 | + | − | nd | − | − | + | − |
| 67 | + | − | nd | − | − | + | − |
| **68** | **+** | **−** | **nd** | **−** | **−** | **−** | **−** |
| **69** | **+** | **−** | **nd** | **−** | **−** | **−** | **−** |
| **70** | **+** | **−** | **+** | **−** | **−** | **+** | **−** |
| 71 | + | − | nd | − | − | + | − |
| **72** | **+** | **+** | **+** | **−** | **−** | **+** | **−** |
| **73** | **+** | **−** | **nd** | **−** | **−** | **−** | **−** |
| **74** | **+** | **−** | **nd** | **−** | **−** | **−** | **−** |
| **75** | **+** | **−** | **nd** | **−** | **−** | **+** | **+** |
| 76 | + | − | nd | − | − | + | − |
| 77 | + | − | nd | − | − | + | − |
| 78 | + | − | nd | − | − | + | − |
| 79 | + | − | nd | − | − | + | − |
| 80 | + | − | nd | − | − | + | − |
| 81 | + | − | nd | − | − | + | − |
| 82 | + | − | nd | − | − | + | − |
| 83 | + | − | nd | − | − | + | − |
| **84** | **+** | **−** | **nd** | **−** | **−** | **−** | **−** |
| 85 | + | − | nd | − | − | + | − |
| 86 | + | − | nd | − | − | + | − |
| **87** | **+** | **−** | **nd** | **−** | **−** | **−** | **−** |
| 88 | + | − | nd | − | − | + | − |
| 89 | + | − | nd | − | − | + | − |
| 90 | + | − | nd | − | − | + | − |
| 91 | + | − | nd | − | − | + | − |
| **92** | **+** | **−** | **nd** | **−** | **−** | **−** | **−** |
| 93 | + | − | nd | − | − | + | − |
| 94 | + | − | nd | − | − | + | − |
| **95** | **+** | **−** | **nd** | **−** | **−** | **−** | **−** |
| 96 | + | − | nd | − | − | + | − |
| 97 | + | − | nd | − | − | + | − |
| 98 | + | − | nd | − | − | + | − |
| 99 | + | − | nd | − | − | + | − |
| 100 | + | − | nd | − | − | + | − |
| **TOTAL** | **96/100** | **6/100** | **6/6 ( gag +)** | **0/100** | **0/100** | **87/100** | **1/100** |
|  |  |  | **1/2 (gag -)** |  |  |  |  |

Rows in bold highlight those samples with results differing from the mode result set. Totals are given at the end of each column for clarity. +, positive result, −, negative result, nd, not determined, na, not applicable. *Hgapdh*, human glyceraldehyde 3-phosphate dehydrogenase, XMRV, xenotropic murine leukaemia virus-related virus, IAP, intra-cisternal A particle, BKV, BK virus, TV, *Trichomonas vaginalis*, hDNA, human DNA, HPV, human papilloma virus.
